# Characterization and comparative genomic analysis of *Pseudaeromonas aegiceratis* sp. nov., a new nitrogen-fixing bacterium from the sediments of mangrove plant *Aegiceras corniculatum*

**DOI:** 10.1128/spectrum.00041-25

**Published:** 2025-03-31

**Authors:** Meng Long, Qiangcai He, Shaoshuai Tang, Zhen Gan, Yishan Lu

**Affiliations:** 1 Guangdong Provincial Engineering Research Center for Aquatic Animal Health Assessment, and Shenzhen Public Service Platform for Evaluation of Marine Economic Animal Seedings, Shenzhen Institute of Guangdong Ocean University 622881, Shenzhen, Guangdong, China; 2 Guangdong Provincial Key Laboratory of Aquatic Animal Disease Control and Healthy Culture, and Key Laboratory of Control for Disease of Aquatic Animals of Guangdong Higher Education Institute, College of Fishery, Guangdong Ocean University 725655, Zhanjiang, Guangdong, China; USDA-ARS-NPRL, Dawson, Georgia, USA

**Keywords:** *Pseudaeromonas*, nitrogen fixation, novel species, complete genome, plant growth-promoting bacteria

## Abstract

A gram-negative, nitrogen-fixing, rod-shaped, and motile bacterium, designated as ZJS20^T^, was isolated from the sediments of *Aegiceras corniculatum* in Zhanjiang, Guangdong, China. The growth of strain ZJS20^T^ occurred at 10°C–45°C, pH 5.5–8.0, and in 0%–3% (wt/vol) NaCl. Strain ZJS20^T^ had the highest sequence similarity of 16S rRNA gene with *Pseudaeromonas paramecia* PCS8^T^ (98.5%), followed by *Pseudaeromonas sharmana* GPTSA-6^T^ (97.1%) and *Pseudaeromonas pectinilytica* AR1^T^ (96.7%). The phylogenetic trees built using 16S rRNA genes and Type (Strain) Genome Server both revealed that strain ZJS20^T^ fell into a cluster with *Pseudaeromonas* sp. strains. The genome of strain ZJS20^T^ has a 3,681,671 bp circular chromosome, a G + C content of 62.7%, and 3,320 proteins-coding genes. The genome contains *nif* and *anf* genes for the synthesis of Mo- and Fe-nitrogenase and genes related to plant growth-promoting traits. Both the digital DNA-DNA hybridization and average nucleotide identity values between strain ZJS20^T^ and type strains of the genus *Pseudaeromonas* were below the cutoff thresholds for species demarcation. The profiles of the predominant fatty acids, major polar lipids, and respiratory quinone of strain ZJS20^T^ were similar to those of other *Pseudaeromonas* species. According to the above results, strain ZJS20^T^ is considered to be a novel species of the genus *Pseudaeromonas*, with the proposed name *Pseudaeromonas aegiceratis* sp. nov. The type strain is ZJS20^T^ (MCCC 1K08760^T^ = KCTC 8214^T^). This study expands the membership of the genus *Pseudaeromonas* and reveals the potential of this genus to be used as plant growth-promoting bacteria with their outstanding nitrogen-fixation ability.

**IMPORTANCE:**

*Pseudaeromonas* is a genus known as close to the genus *Aeromonas* and contains only three species, which were isolated from different sources. Except for the physio-biochemical properties, other characteristics, such as genomic information and functions of the genus, have been reported scarcely to date. Here, the study reports the fourth species (*Pseudaeromonas aegiceratis* ZJS20^T^) to the genus *Pseudaeromonas* and reveals its nitrogen-fixation capacity. Comparative genomic analyses revealed that genomes of strain ZJS20^T^ and other type *Pseudaeromonas* strains all contain *nif* genes (*nifBHDKENXV*) for the synthesis of Mo-nitrogenase, as well as genes related to phosphate metabolism and auxin synthesis. Therefore, we proposed that *Pseudaeromonas* sp. could be considered as a new and potential plant growth-promoting bacteria to be used in agriculture, although in-depth experiments are needed in the future to verify their specific plant growth-promoting traits.

## INTRODUCTION

The genus *Pseudaeromonas*, affiliated with the family *Aeromonadaceae*, phylum *Pseudomonadota*, was first proposed by Padakandla and Chae in 2017 (1). So far, there are only three species described with correct and validly published names (https://lpsn.dsmz.de/genus/pseudaeromonas) in the genus *Pseudaeromonas*. The type species *Pseudaeromonas sharmana* GPTSA-6^T^ was isolated from a warm spring and initially named as *Aeromonas sharmana* GPTSA-6^T^ by Saha and Chakrabarti in 2006 (2). Ever since its description, two studies on the phylogenetic analysis of *Aeromonas* species have revealed the loose phylogenetic relationship of *A. sharmana* GPTSA-6^T^ to members of the genus *Aeromonas*, suggesting that a new genus should be proposed for *A. sharmana* GPTSA-6^T^ (3, 4). However, no new names have been effectively proposed until the strain AR1^T^, showing a close phylogenetic relationship with *A. sharmana* GPTSA-6^T^, was isolated as an ampicillin-resistant bacterium from a freshwater stream in Jeonju, Republic of Korea (1). A polyphasic taxonomy was then conducted by Padakandla and Chae, and the results strengthened the previous observations and also necessitated the description of strain AR1^T^ as a novel taxon. Therefore, *Pseudaeromonas* gen. nov., in the family *Aeromonadaceae*, was proposed, with *P. sharmana* GPTSA-6^T^ as the type species and *Pseudaeromonas pectinilytica* AR1^T^ as the second novel species of the genus (1). *P. paramecii* PCS8^T^, the third novel species of the genus, was isolated from a ciliate *Paramecium caudatum* sample collected from a brackish-water river in Ulsan, Republic of Korea in 2018 (5). The genus *Pseudaeromonas* is gram-negative, facultatively anaerobic, rod-shaped, and motile bacteria, containing ubiquinone-8 as the sole respiratory quinone and summed feature 3, C_16:0_, and summed feature 8 as the predominant polar lipids (Ls) (5).

During the diversity and taxonomy study of bacteria in Gaoqiao mangrove wetlands in Zhanjiang City, Guangdong Province, China, a nitrogen-fixing bacterium designated as ZJS20^T^ was isolated from mangrove plant *Aegiceras corniculatum*’s sediments. In the present study, a polyphasic approach was used to verify the taxonomic status of strain ZJS20^T^, and the results revealed that strain ZJS20^T^ should be identified as the fourth species of the genus *Pseudaeromonas*. The complete genome of strain ZJS20^T^ was sequenced, and comparative genomic analyses among strain ZJS20^T^ and two type strains of the genus *Pseudaeromonas* were conducted, and the results showed the potential of *Pseudaeromonas* sp. as plant growth-promoting bacteria for the presence of a series of genes related to nitrogen fixation, phosphorus metabolism, and auxin synthesis in all the strains.

## MATERIALS AND METHODS

### Bacterial isolation, cultivation, and preservation

Strain ZJS20^T^ was isolated from a sediments sample of the mangrove plant *Aegiceras corniculatum* in Gaoqiao Mangrove Nature Reserve (N21°36'52", E109°47'18") in Zhanjiang City, Guangdong Province, China on 30 October 2019. The soil sample was 10-fold serially diluted and cultured on 2,216 marine agar (MA, Solarbio, China) medium at 28°C for 14 days. Strain ZJS20^T^ was obtained and purified by repeated streaking on MA plates, and then, it was routinely cultured in 2,216 marine broth (MB, Solarbio, China) medium for taxonomy-related experiments and preserved in a glycerol suspension in MB (15%, [vol/vol]) at −80°C. *P. sharmana* DSM 17445^T^ from the Deutsche Sammlung von Mikroorganismen und Zellkulturen and *P. paramecii* KCTC 62038^T^ and *P. pectinilytica* KCTC 42754^T^ from Korean Collection for Type Cultures (KCTC) were used as reference strains for comparative experiments in this study. Strain ZJS20^T^ was deposited in KCTC and Marine Culture Collection of China (MCCC) under KCTC 8214^T^ and MCCC 1K08760^T^, respectively.

### 16S rRNA gene analysis

After cultured in MB medium at 28°C for 48 h, strain ZJS20^T^ genomic DNA was extracted according to the manufacturer’s instruction of a TIANamp Bacteria DNA kit (TIANGEN, China). The 16S rRNA gene of strain ZJS20^T^ was amplified using the genomic DNA as a template and the previously described universal primers for bacteria (6). The PCR products were purified and cloned onto the pMD18-T vector before being sequenced at Sangon Biotech (China). Then, the 16S rRNA gene was uploaded to the EzBioCloud online server (www.ezbiocloud.net) to search for the close relatives of strain ZJS20^T^, and the related 16S rRNA gene sequences in FASTA format were retrieved from EzBioCloud. Subsequently, the alignment of multi-sequences using the Clustal W program, calculation of phylogenetic distances with 1,000 replicates, and construction of phylogenetic trees using neighbor-joining (NJ), maximum-likelihood (ML), and minimum-evolution (ME) methods were performed with MEGA 12 software (7).

### Physiological and biochemical characterization

In the physiological and biochemical experiments, strain ZJS20^T^ and its reference strains were all cultured on MA medium at 28°C for 48 h, unless otherwise stated. Cell morphology was observed and photographed through a transmission electron microscope (TEM, Hitachi, HT7800). Gram staining was examined by using a Gram stain kit (Huankai Microbial, China). Cell motility was detected using the previously described hanging drop method (8). The anaerobic growth was tested according to the growth of strain ZJS20^T^ on MA medium in an anaerobic jar (10% H_2_, 10% CO_2_, and 80% N_2_) at 28°C for a week with or without 0.1% (wt/vol) KNO_3_. Oxidase and catalase reaction activities were detected by using an oxidase test and a catalase detection reagent from Huankai Microbial (China), respectively. Growth at different temperatures (4, 10–30 [in increments of 5], 32–44 [in increments of 2], 45, and 46) was tested on MA medium for 7 days. Growth in NaCl concentrations ([wt/vol], 0%–10% [in increments of 1%], 12%, and 15%) was observed using a medium prepared with distilled water (Na^+^-free, 0.1% yeast extract, and 0.5% peptone). Growth at different pH (from 3.0 to 12.0, at increments of 0.5 units) was examined by adjusting the MB medium using a buffer system as described in our previous articles (9, 10). Furthermore, in order to compare the growth rate and generation time among strain ZJS20^T^ and three reference strains, all four strains were cultured in a medium prepared with distilled water (Na^+^-free, 0.1% yeast extract, and 0.5% peptone) under the tested optimum conditions, and OD_600_ values of the bacterial suspensions were measured by UV spectrophotometer continuously for 24 h. Other biochemical properties were assessed by using the API ZYM kit, API 20NE kit, and API 50CHB kit from BioMérieux (France) and GEN III Microplate (Biolog, Germany).

### Chemotaxonomic analyses

For analysis of cellular fatty acid, strain ZJS20^T^ and three reference strains were cultured in MB medium at 28°C and harvested after incubation for 2 days, and then, the cell mass was extracted using the standard protocol and was identified according to the method described previously by Miller (11). To analyze the respiratory quinone and polar lipid profiles, strain ZJS20^T^ was cultured in MB medium at 28°C and harvested and freeze-dried after incubation for 2 days. The polar lipids were extracted using standard procedures and were analyzed using a two-dimensional thin-layer chromatograph according to the published methods (12, 13). Four different spray reagents, including molybdenum blue, ninhydrin, 1-naphthol-sulfuric acid, and molybdatophosphoric acid, were used to detect phospholipids, aminolipids, glycolipids (GLs), and total lipids, respectively. Respiratory quinones of strain ZJS20^T^ were extracted and purified as previously described (14) and then were determined through high-performance liquid chromatography (15).

### Genomic sequencing and analyses

By using the traditional SDS method (16), the genomic DNA of strain ZJS20^T^ was extracted. The quantity of the DNA was tested by agarose gel electrophoresis, and the quality of the DNA was detected by using Qubit 2.0 Fluorometer (Thermo Scientific). The genome of strain ZJS20^T^ was sent to be sequenced at Nextomics Biosciences Co., Ltd (Wuhan, China) using a combination of third- and second-generation sequencing technologies. The reads obtained from Oxford Nanopore third-generation technology were assembled using Flye v2.9, and the data obtained from MGI second-generation technology were used to check the assembled sequences using Nextpolish v1.4.13 (17, 18). The completeness and contamination of the assembled contigs were evaluated using CheckM v1.2.3 (19). The genome was uploaded to the NCBI database and annotated using the Prokaryotic Genome Annotation Pipeline (PGAP, 4.10) and was annotated using the Rapid Annotation Subsystem Technology (RAST) online server as well (20, 21). Genes related to nitrogen fixation and plant growth-promoting traits were annotated and extracted according to the RAST results. The proteins of the genome were also functionally annotated against the Kyoto Encyclopedia of Genes and Genomes (KEGG) database using KopfamScan v1.2.0 (22) and against the Cluster of Orthologous Groups (COG) using blastp with the *e*-value < 1e-5 (23). The antimicrobial resistance (AMR) genes were predicted by blasting the genome sequence against the Comprehensive Antibiotic Resistance Database with “perfect and strict hit only” and “high-quality” selected (24). The biosynthetic gene clusters (BGCs) were analyzed using the antiSMASH v7.0 web service with default parameters (25). The genomic islands were predicted and annotated using the web server of IslandViewer 4 (26). The phylogenomic tree revealing the position of strain ZJS20^T^ among its closely related type strains was constructed based on the intergenomic distances calculated from the Genome Blast Distance Phylogeny comparison method in the Type (Strain) Genome Server (TYGS) (27). The genomes of strain ZJS20^T^ and two *Pseudaeromonas* type strains available in the NCBI database were included for comparative genomic analyses. The average nucleotide identity (ANI) value was calculated using the ANI calculator with the OrthoANIu algorithm (28), the digital DNA-DNA hybridization (dDDH) value was predicted by the online Genome-to-Genome Distance Calculator 3.0 (27), and orthologous clusters (OCs) were analyzed by using the online server of OrthoVenn 3.0 (29).

## RESULTS AND DISCUSSION

### Phylogenetic relationship

**Fig 1:**
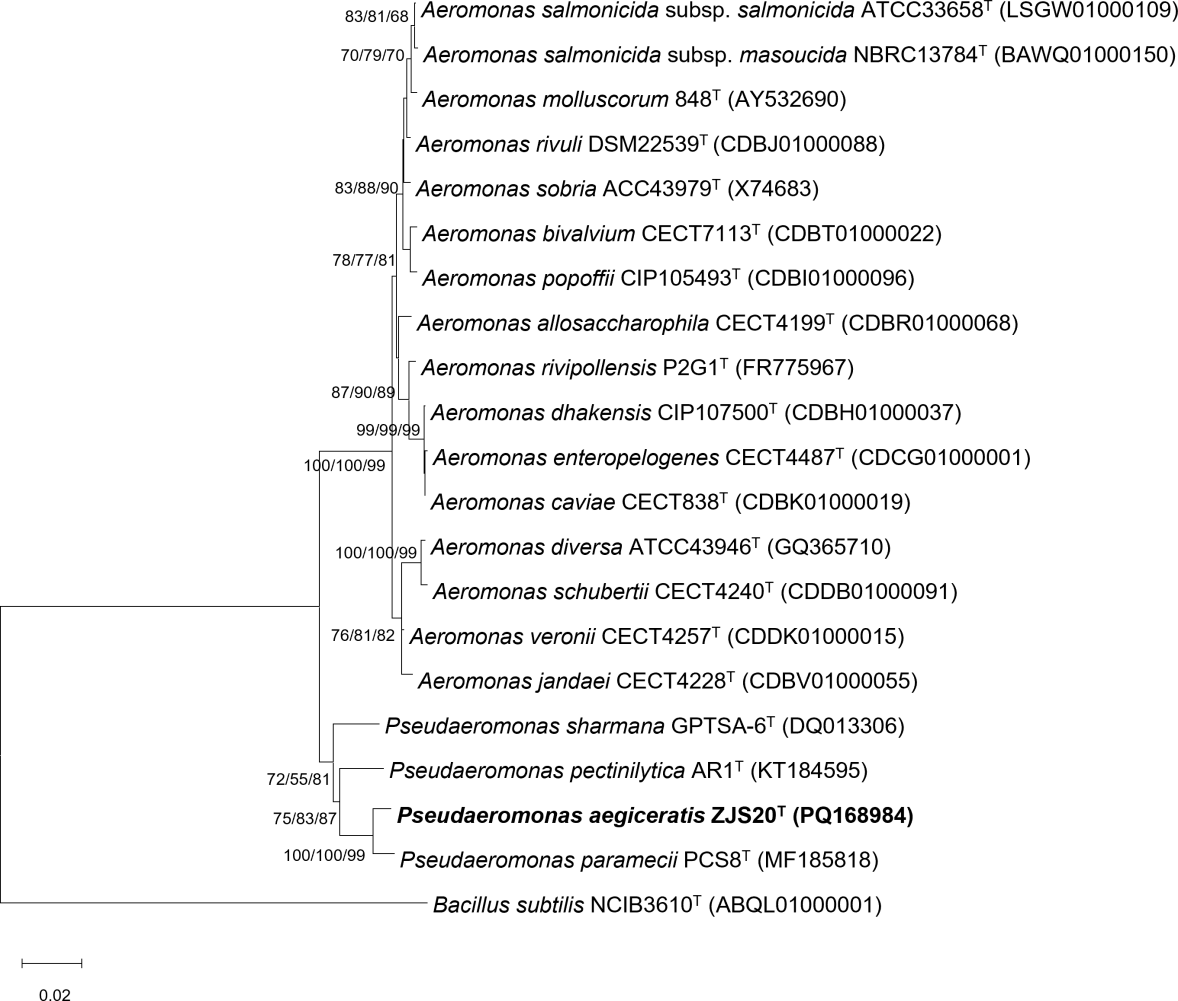
Phylogenetic tree based on the 16S rRNA gene sequences showing the phylogenetic relationships of strain ZJS20^T^ and its closely related species. *Bacillus subtilis* NCIB3610^T^ was used as the outgroup. Bootstrap values referring to NJ/ME/ML analysis are based on 1,000 replicates, and values greater than 70 are shown at branch points. Bar, 0.02 substitution per nucleotide position.

The almost complete sequence of the 16S rRNA gene of strain ZJS20^T^ was 1,465 bp in length. According to the pairwise comparison results of 16S rRNA gene sequences between strain ZJS20^T^ and type strains in the EzBioCloud database, strain ZJS20^T^ shared the highest sequence similarity with *P. paramecii* KCTC 62038^T^ (98.49%), followed by *P. sharmana* DSM 17445^T^ (97.13%) and *P. pectinilytica* KCTC 42754^T^ (96.73%). Members of other genera of the family *Aeromonadaceae* showed a more distant relationship with strain ZJS20^T^, with similarities less than 95%. The NJ phylogenetic tree showed that strain ZJS20^T^ formed an independent cluster with three type strains of the genus *Pseudaeromonas*, with *P. paramecii* KCTC 62038^T^ as its closest relative (Fig. 1). In addition, the ME and ML phylogenetic analyses revealed the similar topological relationship with that of the NJ tree (Fig. 1), and the corresponding bootstrap values were presented on the branches in the order of NJ, ME, and ML.

### Phenotypic, physiological, and biochemical properties

**Fig 2:**
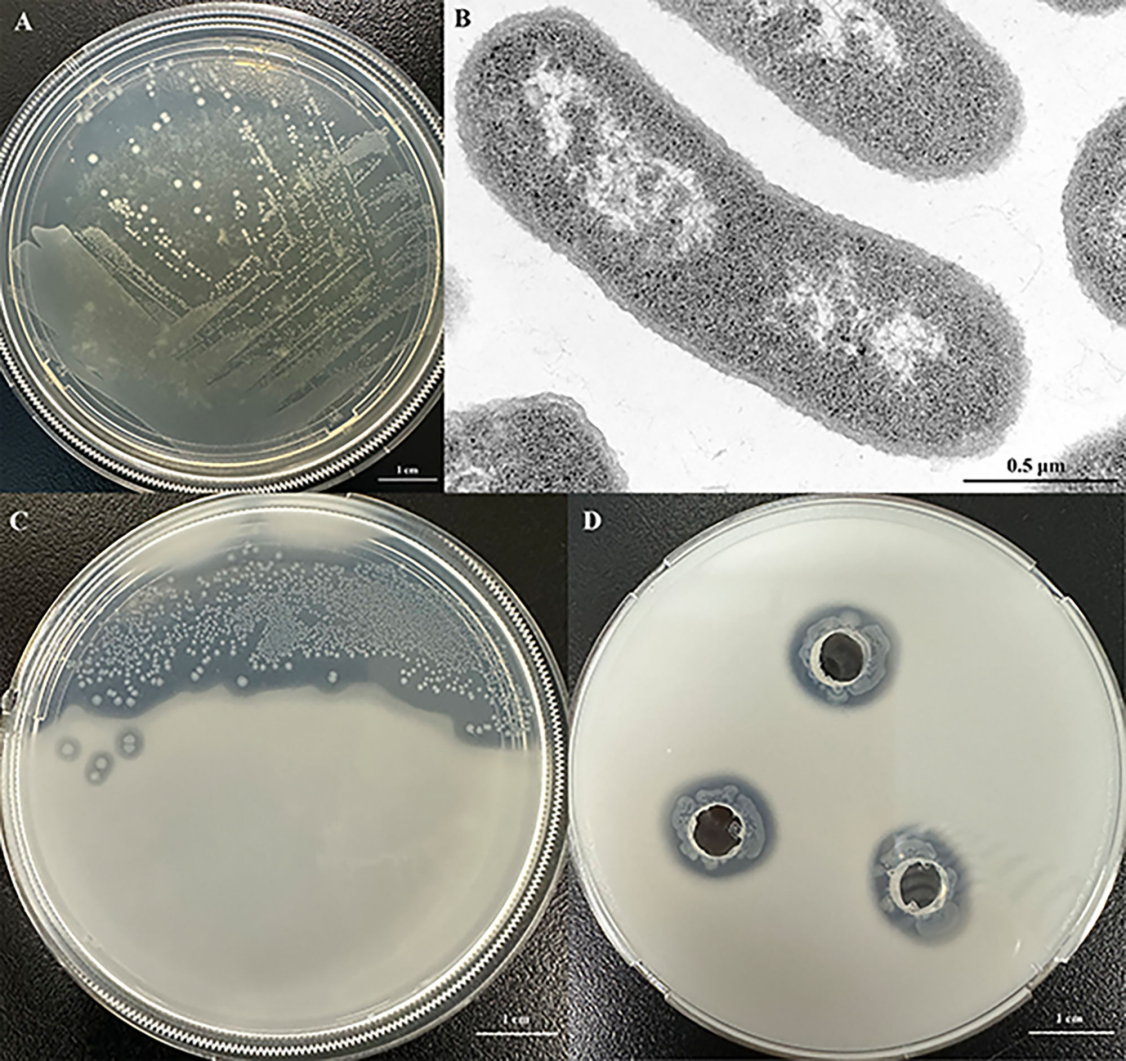
The plate picture showing the colony morphology (**A**) and the transmission electron micrograph (**B**) of strain ZJS20^T^ grown on 2216E agar medium at 28°C for 2 days. The transparent halos around the colony (**C**) or around the hole with culture (**D**) of strain ZJS20^T^ on Ashby’s agar medium, showing the nitrogen-fixation ability of strain ZJS20^T^.

Cells of strain ZJS20^T^ were gram-negative, motile, and facultatively anaerobic. The cells were rod shaped (0.5–0.7 µm × 1.0–2.2 µm) by observation through a TEM (Fig. 2). Colonies of strain ZJS20^T^ after grown on MA medium for 48 h were creamy white, circular, and rough. Strain ZJS20^T^ grew at a temperature range of 10°C–45°C, a pH range of 5.5–8.0, and in 0%–3% NaCl (wt/vol), with optimum growth at 28°C–30°C, pH 7.0, and in 1% NaCl. Growth curves of strain ZJS20^T^ and the three reference strains cultured under the tested optimum conditions (28°C, pH 7, and 1% NaCl) were plotted and presented in Fig. S1. The growth curve of strain ZJS20^T^ was different from those of the three reference strains. In detail, for strain ZJS20^T^, the lag phase and logarithmic phase were 0–1 h and 2–6 h, and the stable phase began at 7 h; while for the three reference strains, the lag phase and logarithmic phase were 0–5 h and 6–10 h, and the stable phase began at 11 h. The generation time of the four strains was calculated according to the formula as previously described (30) and was 55 min, 62 min, 65 min, and 67 min for strain ZJS20^T^, *P. paramecii* KCTC 62038^T^, *P. sharmana* DSM 17445^T^, and *P. pectinilytica* KCTC 42754^T^, respectively. Therefore, compared to the three reference strains, strain ZJS20^T^ grows faster with a relatively short generation time. The oxidase and catalase activity of strain ZJS20^T^ were negative and positive, respectively, while the two activities in three reference strains were both negative. The results of phenotypic, physiological, and biochemical experiments (API 20NE, API ZYM, 50CHB, and GNIII Microplate tests) differentiated strain ZJS20^T^ from three reference strains, as listed in Table 1, and the same test results among them were listed in Table S1. The detailed characteristics of strain ZJS20^T^ were given in the section of species description.

**TABLE 1:** Differential characteristics of strain ZJS20^T^ and the reference strains^*a*^

Characteristics	1	2	3	4
Cell size (μm)	1.0–2.2 × 0.5–0.7	1.5–2.8 × 0.5–1.0^*c*^	1–2 × 0.5^*d*^	1.5–2.5 × 0.5–1.0^*e*^
Flagella and motility	−	−^*d*^	+^*d*^	+^*d*^
Temperature range (optimum) for growth (°C)	10–45 (28-30)	12–44 (36-37)^*c*^	10–45 (28)^*d*^	10–45 (30)^*e*^
pH range (optimum) for growth	5.5–8.0 (7.0)	6.0–10.0 (7.0)^*c*^	6.0–8.0 (7.0–8.0)^*d*^	6.0–8.0 (8.0)^*e*^
NaCl range (optimum) for growth (%, [wt/vol])	0–3.0 (1.0)	0–3.0 (1.0–2.0)^*c*^	0–4.0 (1.0)^*d*^	0–1.5 (1.0)^*e*^
Oxidase	−	−	−	−
Catalase	+	−	−	−
API 20NE test results:				
Assimilation of _D_-glucose, _L_-arabinose, _D_-mannitol, and _D_-maltose	+	−	−	−
API ZYM test results:				
Alkaline phosphatase	−	−	+	−
Esterase and valine arylamidase	W	−	−	−
Esterase lipase	−	W	−	−
Leucine arylamidase	+	+	−	+
Acid phosphatase	+	W	+	+
*β*-galactosidase	W	+	−	−
*α*-glucosidase	+	−	−	+
*β*-glucosidase	+	+	−	−
Utilization of substrates in Biolog GENIII:				
Dextrin, _D_-serine, _D_-maltose, sucrose, *β*-methyl-_D_-glucoside, and _D_-mannose	+	−	+	+
_D_-trehalose	+	−	−	−
_D_-cellobiose	−	−	+	+
_D_-salicin	−	−	W	+
_N_-acetyl-_D_-glucosamine	−	−	+	+
_D_-sorbitol	+	+	−	−
_D_-mannitol	+	−	+	+
Pectin	−	−	W	−
50CHB test results:				
_L_-rhamnose, sorbitol, and _D_-trehalose	+	−	−	−
_L_-arabinose	+	+	−	−
_N_-acetylglucosamine	−	+	+	+
_D_-lactose	−	−	+	+
_D_-gentiobiose	−	−	+	+
_D_-turanose	−	+	−	−
_D_-lyxose	−	W	−	−
Polar lipids	Diphosphatidylglycerol (DPG), phosphatidylglycerol (PG), phosphatidylethanolamine (PE), glycolipid (GL), and lipid (L)	PG, PE, aminolipid (AL), and L^*c*^	DPG, PG, PE, phospholipid (PL1–4), and AL^*d*^	DPG, PG, PE, PL1–4, and aminophospholipid (APL)^*e*^
DNA G + C content (%)^*b*^	62.7	61.5	57.0	54.7

^*a*^ 1, ZJS20^T^; 2, *Pseudaeromonas paramecii* KCTC 62038^T^; 3, *P. sharmana* DSM 17445^T^); 4, *P. pectinilytica* KCTC 42754^T^. +, positive; −, negative; W, weakly positive. All data are from this study unless otherwise indicated.

^*b*^ DNA G + C contents were calculated from the genome data.

^*c*^ Data were taken from Akter et al. (5).

^*d*^ Data were taken from Saha et al. (2).

^*e*^ Data were taken from Padakandla et al. (1).

### Chemotaxonomic characteristics

The predominant fatty acids (>10%) of strain ZJS20^T^ were summed feature 8 (C_18:1_ ω7c; 12.57%), C_16:0_ (32.79%), and summed feature 3 (C_16:1_ ω7c/_C16:1_ ω6c; 33.56%), and the main fatty acids (>1%) of strain ZJS20^T^ were summed feature 2 (iso-C_16:1_ I/C_14:0_ 3-OH; 7.67%), C_14:0_ (6.28%), C_12:0_ (4.1%), and C_18:0_ (1.11%), both of which were similar to those of three reference strains (Table 2). The polar lipid profile of strain ZJS20^T^ was composed of phosphatidylglycerol (PG), diphosphatidylglycerol (DPG), phosphatidylethanolamine (PE), an unidentified GL, and L. Although strain ZJS20^T^ shared polar lipids of DPG, PG, and PE with *P. sharmana* DSM 17445^T^ and *P. pectinilytica* KCTC 42754^T^, it could be distinguished from these two strains by the presence of GL and L and the absence of PL (Fig. S2). The respiratory quinone of strain ZJS10^T^ was the sole ubiquinone-8, which was consistent with other members of the genus *Pseudaeromonas*.

**TABLE 2:** Cellular fatty acid compositions of strain ZJS20^T^ and the reference strains^*a*^

Fatty acid	1	2	3	4
C_12:0_	4.10	4.73	5.24	5.3
C_14:0_	6.28	4.74	4.04	4.8
C_16:0_	**32.79^*c*^**	**31.43^*c*^**	**26.65^*c*^**	**33.6^*c*^**
C_18:0_	1.11	1.11	2.01	1.1
Summed feature 2^*b*^	7.67	9.19	5.76	6.2
Summed feature 3^*b*^	**33.56^*c*^**	**35.63^*c*^**	**28.99^*c*^**	**36.5^*c*^**
Summed feature 8^*b*^	**12.57^*c*^**	8.84	**23.12^*c*^**	**10.2^*c*^**

^*a*^ 1, ZJS20^T^; 2, *Pseudaeromonas paramecii* KCTC 62038^T^; 3, *P. sharmana* DSM 17445^T^; 4, *P. pectinilytica* KCTC 42754^T^. Values are percentages of the total fatty acids. Fatty acids amounting to <1.0% are not shown; dominant fatty acids (≥10.0%) are highlighted in bold.

^*b*^ Summed feature 2 contains iso-C_16:1_ I/C_14:0_ 3-OH; summed feature 3 contains C_16:1_ω7c/C_16:1_ω6c; C_18:2_*ω*6,9c/C_18:0_ ante; summed feature 8 contains C_18:1_ω7c.

^*c*^ Values in bold indicate the dominant fatty acids (≥10.0%).

### Genomic features and functional annotation

**Fig 3:**
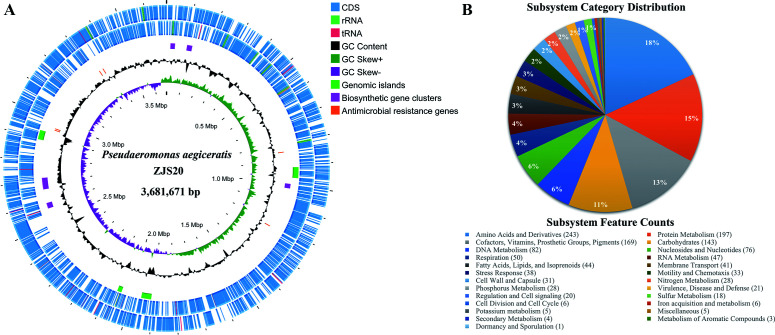
Circular map and subsystem category analysis of strain ZJS20^T^. (**A**) The genomic features of strain ZJS20^T^ are indicated in a circular diagram by seven layers from the innermost to the outermost: GC skew (purple and green), G + C content (black), AMR genes (orange), BGCs (rose red), genomic islands (light green), and CDS on the reverse and forward strand (light blue). (**B**) The subsystem categories of the strain ZJS20^T^ genome were annotated using RAST.

The genome of strain ZJS20^T^ consists of one circular chromosome of 3,681,671 bp, with a G + C content of 62.7% and a sequencing depth of 270×. According to the assessment results from CheckM, the genome is of high quality in terms of completeness (98.2%) and contamination (2.1%). A total of 3,453 genes, 3,320 protein-coding genes (CDSs), 104 tRNAs, and 25 rRNAs (9/8/8 of 5S/16S/23S rRNA genes) were annotated in the genome. A circular map of the genome with its general characteristics was drawn and shown in Fig. 3A. The CDSs were grouped into 25 subsystems based on their involvement in specific biological processes or structural complexes using the RAST annotation tool (Fig. 3B). The CDSs were also annotated and classified into 45 KEGG pathways, among which carbohydrate metabolism, cofactors, vitamins metabolism, etc. were the main pathways (Fig. S3A). The CDSs were annotated and classified into 24 COG functional groups, with signal transduction mechanisms, amino acid transport, metabolism, etc. as the predominant categories (Fig. S3B). Six AMRs were predicted in the ZJS20^T^ genome, including *adeF*, *vanY*, *vanT*, *rsmA*, and two *EF-Tu*, which may contribute to the potential resistance to fluoroquinolone, tetracycline, glycopeptide, and elfamycin antibiotics (Fig. 3A). Five putative BGCs, which are responsible for synthesis of secondary metabolites, including terpene, lassopeptide, hserlactone, thiopeptide, and NRP-metallophore, were identified in the ZJS20^T^ genome (Fig. 3A; Table S2). A total of four genomic islands found in the ZJS20^T^ genome were located in different zones across the genome with lengths ranging from 19,065 bp to 48,611 bp (Fig. 3A). No plasmids or prophages were found in the genome.

### Comparative genomic analyses

**Fig 4:**
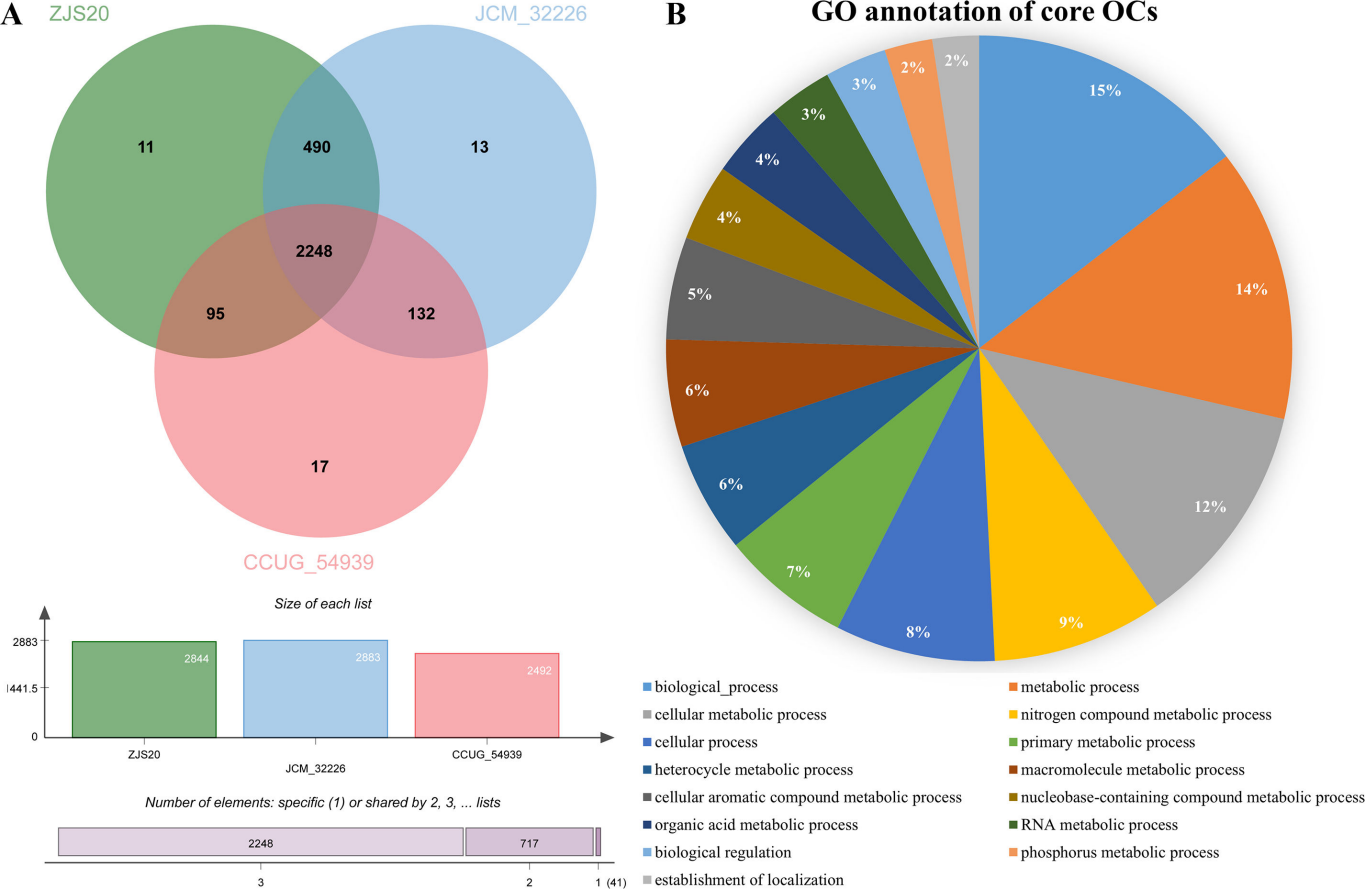
OCs’ analyses by using OrthoVenn 3.0. (**A**) The Venn diagram and bar plots showing the distribution of core, cloud, and unique ortholog clusters among ZJS20^T^, *Pseudaeromonas paramecii* JCM 32226^T^, and *P. sharmana* CCUG 54939^T^, and the cumulative ortholog clusters identified in each of the strains. (**B**) The GO annotation items of the core ortholog clusters, nitrogen compound, and phosphorus metabolic processes are two of the main items.

The phylogenomic tree constructed using TYGS showed that strain ZJS20^T^ fell in a cluster with the two type species within the genus *Pseudaeromonas* (Fig. S4), which confirmed the phylogenetic relationship based on 16S rRNA gene sequences. Therefore, strain ZJS20^T^ was considered to represent a novel member of the genus *Pseudaeromonas*. As shown in Table S3, the ANIb and dDDH values between strain ZJS20^T^ and the two type strains of the genus *Pseudaeromonas* were 74.0%–89.4% and 20.8%–36.9%, respectively, both of which were below the cutoff thresholds of ANI (95%–96%) and dDDH (70%) for demarcation of a novel species (11, 31, 32). According to the results from orthologous clusters’ analyses, a total of 3,006 OCs, including 2,248 core OCs shared by three strains (accounted for 74.8%), 717 accessory OCs shared by two strains (accounted for 23.9%), and 41 unique OCs (accounted for 1.4%), were found among strain ZJS20^T^, *P. paramecii* JCM 32226^T^, and *P. sharmana* CCUG 54939^T^ (Fig. 4A). The core OCs accounted for as high as 74.8%, illustrating the high consistency in certain functions of the genus *Pseudaeromonas*. The gene ontology (GO) annotation results of the core OCs were obtained from OCs’ analyses in OrthoVenn 3.0 and shown in Fig. 4B, which revealed that nitrogen compound and phosphorus metabolic processes were two main GO items among the top 15 items listed. The data suggested that nitrogen fixation and phosphorus metabolism may be the common activities in *Pseudaeromonas* sp. strains. Furthermore, the accessory and unique OCs could be helpful in distinguishing different species from each other in the genus *Pseudaeromonas*.

As shown in Table S2, the number of BGCs predicted in strain ZJS20^T^, *P. paramecii* JCM 32226^T^, and *P. sharmana* CCUG 54939^T^ were 5, 4, and 5, respectively, and among them, BGCs for the synthesis of terpene and thiopeptide were commonly found in three strains. Terpenes produced by bacteria have been proven to be involved in growth promotion, yield increase, and stress resistance of plants (33), and thiopeptides are one of the most complex classes of RiPPs and are antibiotics with activity against several gram-positive pathogens (34). It is worth noting that the similarities between the BGCs predicted in ZJS20^T^, *P. paramecii* JCM 32226^T^, and *P. sharmana* CCUG 54939^T^ and the best-known BGCs were all below 50%, which indicates the potential for discovering novel secondary metabolites in these strains and possible application of the metabolites in agriculture or biotechnology in the future.

According to the results from RAST analyses, 17 genes related to nitrogen fixation, 7 genes related to ammonia assimilation, 4 genes related to auxin synthesis, and 10 genes associated with phosphorus metabolism were annotated and found in the ZJS20^T^ genome, and the detailed information was listed in Table 3. Nitrogen fixation of bacteria is catalyzed by nitrogenases, including Mo-, Fe-, and V-nitrogenase, which are encoded accordingly by *nif*, *anf*, and *vnf* genes (35, 36). Strain ZJS20^T^ consisted of *nif* genes (*nifBHDKENXV*) and *anf* genes (*VanfHDGK*) responsible for the synthesis of Mo- and Fe-nitrogenase, respectively, illustrating its strong potential in nitrogen fixation. In addition, the nitrogen-fixation function has been verified by observing the formation of transparent halos around the colony or holes with cultures of strain ZJS20^T^ on Ashby’s agar medium (Fig. 2C and D). In addition, genes associated with ammonia assimilation, such as *glnA*, *glnD*, *glnE*, and *ptsN*, were detected in the ZJS20^T^ genome. Auxins, mainly indole-3-acetic acid (IAA), are a kind of phytohormones, that are involved in regulating the metabolism and growth of plants (37). Several genes related to IAA production, such as *trp*ABDCF, were identified in the ZJS20^T^ genome (Table 3). Phosphorus is one of the essential nutrients for plant growth and development, and phosphate metabolism-related genes, such as *ppk*, *pho*, etc., were detected in the ZJS20^T^ genome (Table 3). Specifically, *ppk1*, *ppk2*, *ppgK*, *ppx*, and *gppA* contribute to polyphosphate synthesis, and *phoB*, *phoH*, *phoR*, and *phoU* are involved in phosphate transport and regulation. In order to verify whether *P. paramecii* JCM 32226^T^ and *P. sharmana* CCUG 54939^T^ contain genes related to nitrogen fixation and plant growth-promoting traits like that of strain ZJS20^T^, genomes of the two strains were also uploaded and annotated using the RAST server. The results revealed that *P. sharmana* CCUG 54939^T^ contained *nif* and *vnf* genes for the synthesis of Mo- and Fe-nitrogenase, while *P. paramecii* JCM 32226^T^ only contained *nif* genes for the synthesis of Mo-nitrogenase. It is speculated that Fe-nitrogenase may function as a complementary nitrogenase but not an alternative enzyme to Mo-nitrogenase in *Pseudaeromonas* sp. strains, and this has been validated in other bacteria like *Rhodobacter capsulatus* (38). Furthermore, both strains contained the related genes for ammonia assimilation, auxin synthesis, and phosphate metabolism (Tables S4 and S5). Therefore, the presence of these gene signatures within ZJS20^T^, as well as in the other two type strains of the genus *Pseudaeromonas*, has revealed the potential of *Pseudaeromonas* sp. as an important plant growth-promoting bacteria that can improve soil fertility through nitrogen, phosphorus, and auxin to its host, although further experiments in future research are needed to verify their specific functions, such as IAA production and phosphorus solubilization.

**TABLE 3:** The nitrogen fixation and plant growth-promoting traits related genes and their functional roles in strain ZJS20^T^ genome annotated by RAST (Rapid Annotation using Subsystem technology)

Genes	Accession no.	Functional role
Nitrogen fixation		
nifV	ABHF91_05670	Homocitrate synthase
nifD	ABHF91_05575	Nitrogenase (molybdenum-iron) alpha chain
–^*a*^	ABHF91_06880	4Fe-4S ferredoxin, nitrogenase-associated
nifA	ABHF91_05900	Nitrogenase (molybdenum-iron)-specific transcriptional regulator NifA
anfK	ABHF91_14630	Nitrogenase (iron-iron) beta chain
anfH	ABHF91_14615	Nitrogenase (iron-iron) reductase and maturation protein AnfH
nifE	ABHF91_05595	Nitrogenase FeMo-cofactor scaffold and assembly protein NifE
anfD	ABHF91_14640	Nitrogenase (iron-iron) alpha chain
nifH	ABHF91_14645	Nitrogenase (molybdenum-iron) reductase and maturation protein NifH
nifB	ABHF91_05915	Nitrogenase FeMo-cofactor synthesis FeS core scaffold and assembly protein NifB
nifN	ABHF91_05600	Nitrogenase FeMo-cofactor scaffold and assembly protein NifN
nifK	ABHF91_05580	Nitrogenase (molybdenum-iron) beta chain
nifX	ABHF91_05590	Nitrogenase FeMo-cofactor carrier protein NifX
nifQ	ABHF91_05920	Nitrogenase FeMo-cofactor synthesis molybdenum delivery protein NifQ
anfG	ABHF91_14635	Nitrogenase (iron-iron) delta chain (EC 1.18.6.1)
nifU	ABHF91_05635	Iron-sulfur cluster assembly scaffold protein NifU
Ammonia assimilation		
–^*a*^	ABHF91_15210	Ammonium transporter
–^*a*^	ABHF91_04795	Glutamate synthase (NADPH) large chain
ptsN	ABHF91_14385	Nitrogen regulatory protein P-II
–^*a*^	ABHF91_04800	Glutamate synthase (NADPH) small chain
glnA	ABHF91_16195	Glutamine synthetase type I
glnE	ABHF91_15455	Glutamate-ammonia-ligase adenylyltransferase
glnD	ABHF91_12400	Uridylyltransferase
Auxin synthesis		
trpA	ABHF91_11320	Tryptophan synthase alpha chain
trpB	ABHF91_10005	Tryptophan synthase beta chain
trpD	ABHF91_11305	Anthranilate phosphoribosyltransferase
trpCF	ABHF91_11310	Phosphoribosylanthranilate isomerase
Phosphate metabolism		
phoU	ABHF91_06970	Phosphate transport system regulatory protein PhoU
phoB	ABHF91_06900	Phosphate regulon transcriptional regulatory protein PhoB (SphR)
phoR	ABHF91_06905	Phosphate regulon sensor protein PhoR (SphS)
phoH	ABHF91_04235	Predicted ATPase related to phosphate starvation-inducible protein PhoH
–^*a*^	ABHF91_03655	Alkaline phosphatase
ppk1	ABHF91_06950	Polyphosphate kinase
ppk2	ABHF91_00325	Polyphosphate kinase 2
ppx	ABHF91_06945	Exopolyphosphatase
ppgK	ABHF91_03835	Polyphosphate glucokinase
gppA	ABHF91_16445	Guanosine-5'-triphosphate and 3'-diphosphate pyrophosphatase

^*a*^ The en dash means no gene name was retrived from the RAST.

### Emended description of the genus *Pseudaeromonas*

The properties are in line with those of the type species *P. sharmana*, first described by Padakandla and Chae (1). The amendments are as follows: the genome of the genus *Pseudaeromonas* is 3.5–3.7 Mb in size, with a G + C content of 57.0%–62.7%. Strains of this genus contain *nif* genes for the synthesis of Mo-nitrogenase and therefore possess nitrogen-fixation potential.

### Description of *Pseudaeromonas aegiceratis* sp. nov.

#### Pseudaeromonas aegiceratis (ae.gi.ce.ra'tis. N.L. gen. n. aegiceratis of mangrove plant Aegiceras corniculatum)

Cells are gram-negative, facultatively anaerobic, motile, and rod shaped (1.0–2.2 µm long and 0.5–0.7 µm wide). The colony is creamy white, circular, and rough after being grown on 2,216 marine agar medium at 28°C for 2 days. Growth is observed at a temperature of 10°C–45°C (optimum 28°C–3 °C), in 0%–3% NaCl ([wt/vol], optimum 1%), and at a pH range of 5.5–8.0 (optimum 7.0). The generation time is 55 min under the optimum conditions. The catalase activity is positive, but the oxidase activity is negative. According to the results of API 20NE tests, _D_-glucose fermentation, esculin hydrolysis, and assimilation of arabinose, glucose, and mannitol are positive, and other tests are negative. For API ZYM tests, acid phosphatase, leucine arylamidase, *α*- and *β*-glucosidase, and naphthol-AS-BI-phosphohydrolase are positive, esterase (C4), *β*-galactosidase, and valine arylamidase are weakly positive, and other tests are negative. According to the results from GEN III MicroPlate tests, utilization of _D_-maltose, _D_-trehalose, sucrose, dextrin, *β*-methyl-_D_-glucoside, _D_-mannose, *α*-_D_-glucose, _D_-fructose, _D_-sorbitol, and _D_-mannitol are positive, and other tests are negative. For 50CHB tests, assimilation of _L_-arabinose, _D_-galactose, _D_-fructose, _D_-glucose, _D_-mannose, _L_-rhamnose, sorbitol, mannitol, methyl-*α*-_D_-glucopyranoside, salicin, esculin, _D_-cellobiose, _D_-maltose, _D_-trehalose, _D_-sucrose, glycogen, starch, and potassium-2-ketogluconate are positive, and other reactions are negative. The predominant cellular fatty acids (>10%) consist of C_16:0_ (32.79%), summed feature 3 (C_16:1_ ω7c/_C16:1_ ω6c; 33.56%) and summed feature 8 (C_18:1_ ω7c; 12.57%). The polar lipids profile comprises phosphatidylglycerol, diphosphatidylglycerol, phosphatidylethanolamine, an unidentified glycolipid, and lipid. The respiratory quinone is the sole ubiquinone-8.

The type strain, ZJS20^T^ (MCCC 1K08760^T^ = KCTC 8214^T^), was isolated from the sediments of mangrove plant *Aegiceras corniculatum*, which was collected from Gaoqiao mangrove wetlands in Zhanjiang, Guangdong, China. The genome of strain ZJS20^T^ is 3,681,671 bp in size and the G + C content is 62.7%.

## Data Availability

In the NCBI GenBank database, the 16S rRNA gene and complete genome sequences of strain ZJS20^T^ were uploaded and registered as PQ168984 and CP158016 for accession, respectively.

## SUPPLEMENTAL MATERIAL

Supplemental material spectrum.00041-25-s0001.docx Supplemental tables and figures.

ASM does not own the copyrights to Supplemental Material that may be linked to, or accessed through, an article. The authors have granted ASM a non-exclusive, world-wide license to publish the Supplemental Material files. Please contact the corresponding author directly for reuse.
