# Supplementary material for: Characterization and comparative genomic analysis of Pseudaeromonas aegiceratis sp. nov., a new nitrogen-fixing bacterium from the sediments of mangrove plant Aegiceras corniculatum
Source: Microbiol Spectr. 2025 Mar 31;13(5):e00041-25. doi: 10.1128/spectrum.00041-25 (PMC12054148; doi:10.1128/spectrum.00041-25)

**Supplementary Table 1.** The same characteristics of API 20NE, API ZYM and Biolog GNIII tests among strains ZJS20^T^ and three type strains of the genus *Pseudaeromonas*. 1, ZJS20^T^; 2, *P. paramecii* KCTC 62038^T^; 3, *P. sharmana* DSM 17445^T^; 4, *P. pectinilytica* KCTC 42754^T^. +, Positive; -, negative; W, weakly positive.

| **Characteristics** | **1** | **2** | **3** | **4** |
| --- | --- | --- | --- | --- |
| API 20NE test results: |  |  |  |  |
| Reduction of nitrates to nitrogen, indole production, arginine dihydrolase, urease, hydrolysis of gelatin, assimilation of _D_-mannose, *N*-acetyl-glucosamine, potassium gluconate, capric acid, adipic acid, malic acid, trisodium citrate and phenylacetic acid | - | - | - | - |
| Fermentation of _D_-glucose, hydrolysis of esculin, *β*-galactosidase | + | + | + | + |
| API ZYM test results: |  |  |  |  |
| Lipase (C14), cystine arylamidase, trypsin, *α*-chymotrypsin, *α*-galactosidase, *β*-glucuronidase, *N*-acetyl-*β*-glucosaminidase, *α*-mannosidase, *α*-fucosidase | - | - | - | - |
| Naphtol-AS-BI-phosphohydrolase | + | + | + | + |
| Biolog GNIII test results: |  |  |  |  |
| Utilization of gentiobiose, _D_-turanose, stachyose, _D_-raffinose, *α*-_D_-lactose, _D_-melibiose, *N*-acetyl-*β*-dmannosamine, *N*-acetyl-_D_-galactosamine, *N*-acetylNeuraminic acid, *N*-acetylNeuraminic acid, 3-methyl glucose, _D_-fucose, _L_-fucose, _L_-rhamnose, _D_-arabitol, myo-inositol, glycerol, _D_-glucose-6-PO_4_, _D_-aspartic acid, _D_-serine, gelatin, glycyl-_L_-proline, _L_-alanine, _L_-arginine, _L_-aspartic acid, L-glutamic acid, _L_-histidine, _L_-pyroglutamic acid, _L_-serine, _D_-galacturonic acid, _L_-galactonic acid lactone, _D_-gluconic acid, _D_-glucuronic acid, glucuronamide, mucic acid, quinic acid, _D_-saccharic acid, p-hydroxy-phenylacetic acid, methyl pyruvate, _D_-lactic acid methyl ester, _L_-lactic acid, citric acid, α-keto-glutaric acid, _D_-malic acid, _L_-malic acid, bromo-succinic acid, tween 40, *γ*-amino-butryricacid, *α*-hydroxy-butyric acid, *β*-hydroxy-_D,L_-butyricacid, *α*-keto-butyric acid, acetoacetic acid, propionic acid, acetic acid, formic acid | - | - | - | - |
| Utilization of *α*-_D_-glucose, _D_-fructose | + | + | + | + |
| 50CHB test results: |  |  |  |  |
| Fermentation of _D_-galactose, _D_-glucose, _D_-fructose, _D_-mannose, mannitol, methyl-*α*-_D_-glucopyranoside, esculin, salicin, _D_-cellobiose, _D_-maltose, _D_-sucrose, starch, glycogen, potassium-2-ketogluconate | + | + | + | + |
| Fermentation of mannitol, erythritol, _D_-arabinose, _D_-ribose, _D_-xylose, _D_-adunculol 1, methyl-β-_D_-xylopyranoside, _L_-sorbitol, dulcitol, inositol, methyl-*α*-_D_-mannopyranoside, amygdalin, arbulin, _D_-melibiose, inulin, _D_-melezitose, _D_-raffinose, xylitol, _D_-tagatose, _D_-fucose, _L_-fucose, _D_-arabinol, _L_-arabinol, potassium gluconate, potassium 5-ketogluconate | - | - | - | - |

**Supplementary Table 2.** The distribution of biosynthetic gene clusters in strain ZJS20^T^ revealed by antiSMASH v7.0. NRP, non-ribosomal peptide; NRPS, non-ribosomal synthesized peptide.

| **Contig** | **Type** | **From** | **To** | **Most similar known cluster** | **Similarity** |
| --- | --- | --- | --- | --- | --- |
| *Pseudaeromonas aegiceratis* ZJS20^T^ | | | | | |
| 1 | Terpene | 18,782 | 39,840 | - | - |
| 1 | Lassopeptide | 97,493 | 121,939 | Potashchelin, NRP | 6% |
| 1 | Hserlactone | 994,197 | 1,015,330 | - | - |
| 1 | Thiopeptide | 2,569,823 | 2,595,936 | - | - |
| 1 | NRP-metallophore, NRPS | 2,661,967 | 2,716,044 | [griseobactin,](https://mibig.secondarymetabolites.org/go/BGC0000640/1) NRP | 38% |
| *Pseudaeromonas paramecii* JCM 32226^T^ | | | | | |
| 8 | Thiopeptide | 194,647 | 220,773 | - | - |
| 16 | Hserlactone | 61,814 | 82,953 | - | - |
| 17 | RiPP-like | 17,502 | 28,368 | - | - |
| 27 | Terpene | 150,344 | 161,556 | - | - |
| *Pseudaeromonas* *sharmana* CCUG 54939^T^ | | | | | |
| 3 | Terpene | 1 | 11,385 | - | - |
| 3 | Butyrolactone | 130,724 | 141,479 | - | - |
| 7 | Arylpolyene | 207,576 | 251,192 | APE Vf | 50% |
| 13 | RiPP-like | 693,991 | 704,848 | - | - |
| 14 | Thiopeptide | 259,539 | 285,658 | - | - |

**Supplementary Table 3.** The 16S rRNA similarity, Average Nucleotide Identity (ANI) value and digital DNA-DNA Hybridization (dDDH) value between strain ZJS20^T^ and the two type strains of the genus *Pseudaeromonas* available in the NCBI database.

| **Strains** | **Genome accession no.** | **16S rRNA similarity (%)** | **ANIb (%)** | **dDDH (%)** |
| --- | --- | --- | --- | --- |
| *P. paramecii* JCM 32226^T^ | BAABFC010000000 | 98.6 | 89.4 | 36.9 |
| *P. sharmana* CCUG 54939^T^ | JBHSAF010000000 | 97.2 | 74.0 | 20.8 |

**Supplementary Table 4.** The nitrogen fixation and other plant growth-promoting traits related genes and their functional roles in strain *Pseudaeromonas paramecii* JCM 32226^T^ genome annotated by RAST (Rapid Annotation using Subsystem technology).

| **Genes** | **Accession No.** | **Functional Role** |
| --- | --- | --- |
| **Nitrogen fixation** | | |
| nifN | ABD847_RS07645 | Nitrogenase FeMo-cofactor scaffold and assembly protein NifN |
| nifB | ABD847_RS12420 | Nitrogenase FeMo-cofactor synthesis FeS core scaffold and assembly protein NifB |
| nifX | ABD847_RS10545 | Nitrogenase FeMo-cofactor carrier protein NifX |
| nifK | ABD847_RS07625 | Nitrogenase (molybdenum-iron) beta chain (EC 1.18.6.1) |
| nifU | ABD847_RS07685 | Iron-sulfur cluster assembly scaffold protein NifU |
| nifQ | ABD847_RS12415 | Nitrogenase FeMo-cofactor synthesis molybdenum delivery protein NifQ |
| nifD | ABD847_RS07620 | Nitrogenase (molybdenum-iron) alpha chain (EC 1.18.6.1) |
| nifV | ABD847_RS07720 | Homocitrate synthase (EC 2.3.3.14) |
| - | ABD847_RS05220 | 4Fe-4S ferredoxin, nitrogenase-associated |
| nifA | ABD847_RS12435 | Nitrogenase (molybdenum-iron)-specific transcriptional regulator NifA |
| nifW | ABD847_RS07725 | Nitrogenase stabilizing/protective protein NifW |
| nifE | ABD847_RS07640 | Nitrogenase FeMo-cofactor scaffold and assembly protein NifE |
| nifH | ABD847_RS07615 | Nitrogenase (molybdenum-iron) reductase and maturation protein NifH |
| **Ammonia assimilation** | | |
| - | ABD847_RS03575 | Ammonium transporter |
| - | ABD847_RS12800 | Glutamate synthase [NADPH] large chain |
| ptsN | ABD847_RS08605 | Nitrogen regulatory protein P-II |
| - | ABD847_RS12805 | Glutamate synthase [NADPH] small chain |
| glnA | ABD847_RS16335 | Glutamine synthetase type I |
| glnE | ABD847_RS11135 | Glutamate-ammonia-ligase adenylyltransferase |
| glnD | ABD847_RS06685 | uridylyltransferase |
| **Auxin synthesis** | | |
| trpA | ABD847_RS05580 | Tryptophan synthase alpha chain |
| trpB | ABD847_RS05575 | Tryptophan synthase beta chain |
| trpD | ABD847_RS05565 | Anthranilate phosphoribosyltransferase |
| trpCF | ABD847_RS05570 | Phosphoribosylanthranilate isomerase |
| **Phosphate metabolism** | | |
| phoU | ABD847_RS05310 | Phosphate transport system regulatory protein PhoU |
| phoB | ABD847_RS05245 | Phosphate regulon transcriptional regulatory protein PhoB |
| phoR | ABD847_RS05250 | Phosphate regulon sensor protein PhoR (SphS) |
| phoH | ABD847_RS13575 | Predicted ATPase related to phosphate starvation-inducible protein PhoH |
| - | ABD847_RS10050 | Alkaline phosphatase |
| ppk1 | ABD847_RS05290 | Polyphosphate kinase 1 |
| ppk2 | ABD847_RS03200 | Polyphosphate kinase 2 |
| ppx | ABD847_RS05285 | Exopolyphosphatase |
| gppA | ABD847_RS03355 | guanosine-5'-triphosphate,3'-diphosphate diphosphatase |

**Supplementary Table 5.** The nitrogen fixation and plant growth-promoting traits related genes and their functional roles in strain *Pseudaeromonas* *sharmana* CCUG 54939^T^ genome annotated by RAST (Rapid Annotation using Subsystem technology).

| **Genes** | **Accession No.** | **Functional Role** |
| --- | --- | --- |
| **Nitrogen fixation** | | |
| nifE | ACFOSS_RS02440 | Nitrogenase FeMo-cofactor scaffold and assembly protein NifE |
| nifH | ACFOSS_RS10400 | Nitrogenase (molybdenum-iron) reductase and maturation protein NifH |
| anfD | ACFOSS_RS10405 | Nitrogenase (iron-iron) alpha chain (EC 1.18.6.1) |
| nifA | ACFOSS_RS02575 | Nitrogenase (molybdenum-iron)-specific transcriptional regulator NifA |
| - | ACFOSS_RS14370 | 4Fe-4S ferredoxin, nitrogenase-associated |
| anfH | ACFOSS_RS10430 | Nitrogenase (iron-iron) reductase and maturation protein AnfH |
| anfK | ACFOSS_RS10415 | Nitrogenase (iron-iron) beta chain (EC 1.18.6.1) |
| nifD | ACFOSS_RS02420 | Nitrogenase (molybdenum-iron) alpha chain (EC 1.18.6.1) |
| nifV | ACFOSS_RS02495 | Homocitrate synthase (EC 2.3.3.14) |
| nifU | ACFOSS_RS02485 | Iron-sulfur cluster assembly scaffold protein NifU |
| anfG | ACFOSS_RS10410 | Nitrogenase (iron-iron) delta chain (EC 1.18.6.1) |
| nifQ | ACFOSS_RS02595 | Nitrogenase FeMo-cofactor synthesis molybdenum delivery protein NifQ |
| nifT | ACFOSS_RS02430 | NifT protein |
| nifX | ACFOSS_RS02435 | Nitrogenase FeMo-cofactor carrier protein NifX |
| nifK | ACFOSS_RS02425 | Nitrogenase (molybdenum-iron) beta chain (EC 1.18.6.1) |
| nifN | ACFOSS_RS02445 | Nitrogenase FeMo-cofactor scaffold and assembly protein NifN |
| nifB | ACFOSS_RS02590 | Nitrogenase FeMo-cofactor synthesis FeS core scaffold and assembly protein NifB |
| nifZ | ACFOSS_RS02505 | NifZ protein |
| **Ammonia assimilation** | | |
| amt | ACFOSS_RS03755 | Ammonium transporter |
| - | ACFOSS_RS16120 | Glutamate synthase [NADPH] large chain |
| ptsN | ACFOSS_RS09695 | Nitrogen regulatory protein P-II |
| - | ACFOSS_RS16125 | Glutamate synthase [NADPH] small chain |
| glnA | ACFOSS_RS08115 | Glutamine synthetase type I |
| glnE | ACFOSS_RS14930 | Glutamate-ammonia-ligase adenylyltransferase |
| glnD | ACFOSS_RS15695 | uridylyltransferase |
| **Auxin synthesis** | | |
| trpA | ACFOSS_RS13860 | Tryptophan synthase alpha chain |
| trpB | ACFOSS_RS10885 | Tryptophan synthase beta chain |
| trpD | ACFOSS_RS13845 | Anthranilate phosphoribosyltransferase |
| trpCF | ACFOSS_RS13850 | Phosphoribosylanthranilate isomerase |
| **Phosphate metabolism** | | |
| phoU | ACFOSS_RS14230 | Phosphate transport system regulatory protein PhoU |
| phoB | ACFOSS_RS14290 | Phosphate regulon transcriptional regulatory protein PhoB |
| phoR | ACFOSS_RS14285 | Phosphate regulon sensor protein PhoR (SphS) |
| phoH | ACFOSS_RS00420 | Predicted ATPase related to phosphate starvation-inducible protein PhoH |
| - | ACFOSS_RS14970 | Alkaline phosphatase |
| ppk1 | ACFOSS_RS14250 | Polyphosphate kinase 1 |
| ppk2 | ACFOSS_RS12610 | Polyphosphate kinase 2 |
| ppx | ACFOSS_RS14255 | Exopolyphosphatase |
| gppA | ACFOSS_RS08655 | guanosine-5'-triphosphate,3'-diphosphate diphosphatase |
| ppgK | ACFOSS_RS06635 | Polyphosphate glucokinase |

**Supplementary Figure 1**. Growth curves of strain ZJS20^T^ and the three type strains under the tested optimum conditions (28 °C, pH 7.0 and 1% (w/v) NaCl) by measuring the optical density at 600 nm. All the curves were done in triplicate with the standard error bars shown. ZJS20^T^, *Pseudaeromonas aegiceratis* ZJS20^T^; KCTC 62038^T^, *P. paramecii* KCTC 62038^T^; DSM 17445^T^, *P. sharmana* DSM 17445^T^; KCTC 42754^T^, *P. pectinilytica* KCTC 42754^T^.


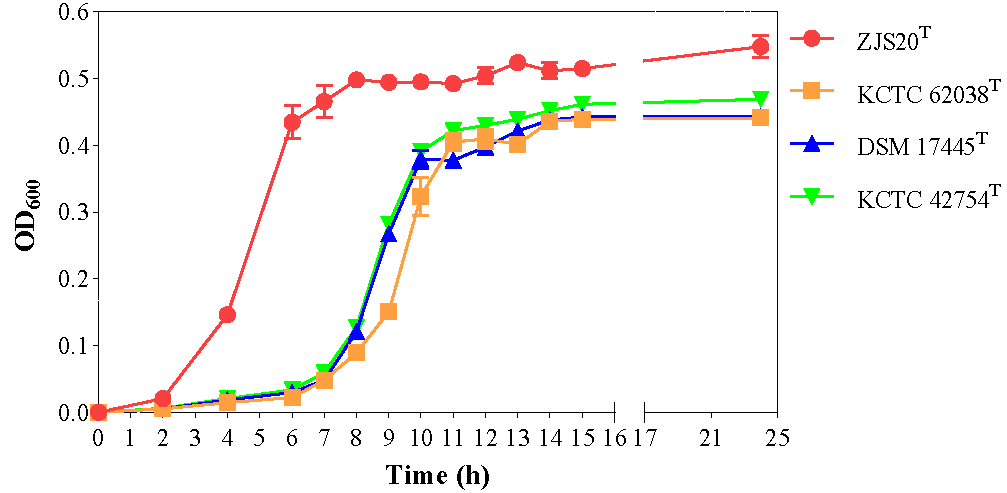


**Supplementary Figure 2**. Polar lipid profile of strain ZJS20^T^. Total lipids (A), phospholipids (B), aminolipids (C) and glycolipids (D) were detected by using reagents of molybdatophosphoric acid, molybdenum blue (0.2%, w/v), ninhydrin and 1-naphthol-sulphuric acid, respectively. DPG: diphosphatidylglycerol; PE: phosphatidylethanolamine; PG: phosphatidylglycerol; GL: one unidentified glycolipid; L: one unknown lipid.


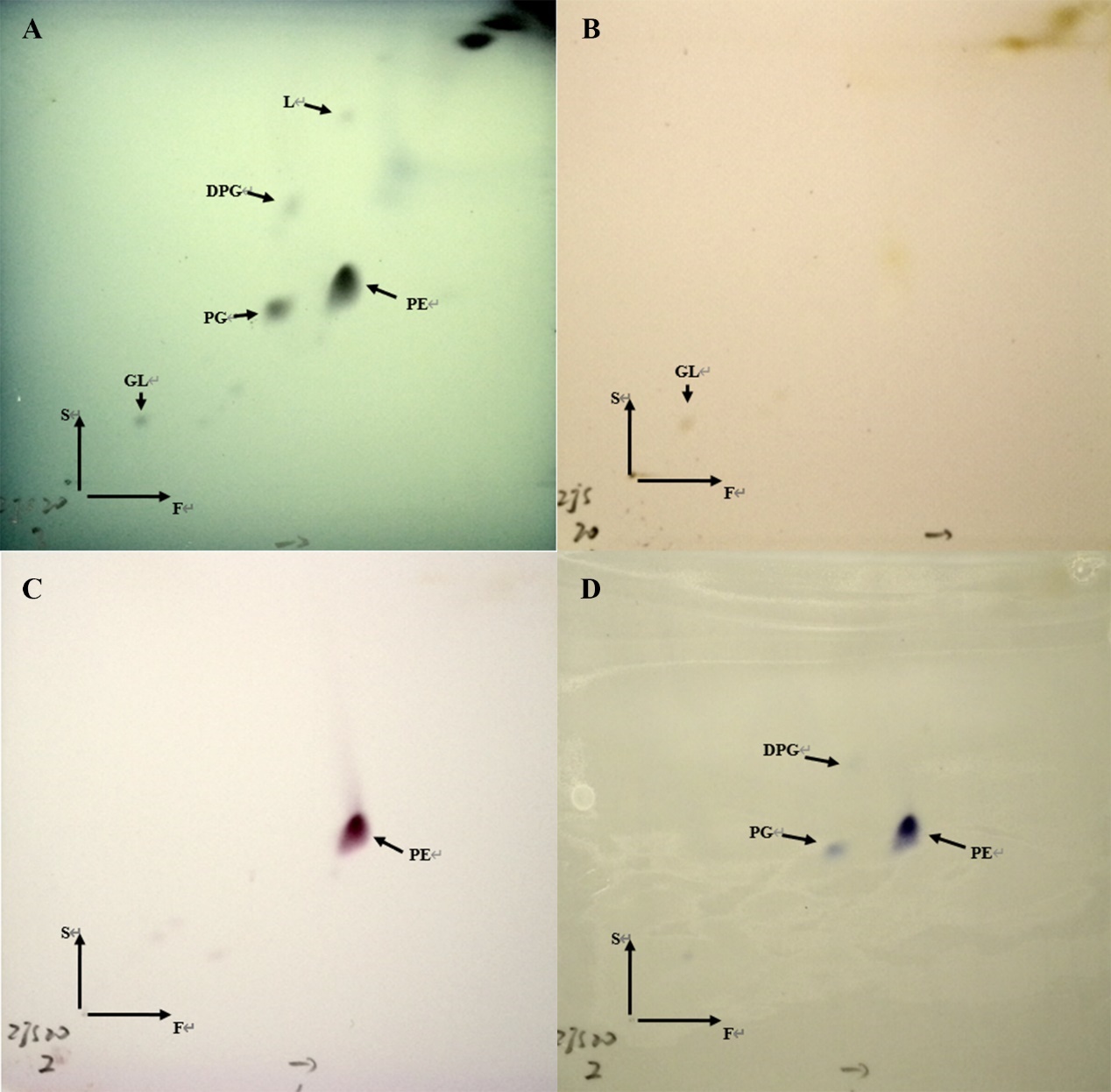


**Supplementary Figure 3**. Functional annotation of ZJS20^T^ genome. The KEGG (A) and COG (B) functional categories in the genome of strain ZJS20^T^. The functional categories are listed in Y axis and the number of genes for corresponding categories are listed in X axis.


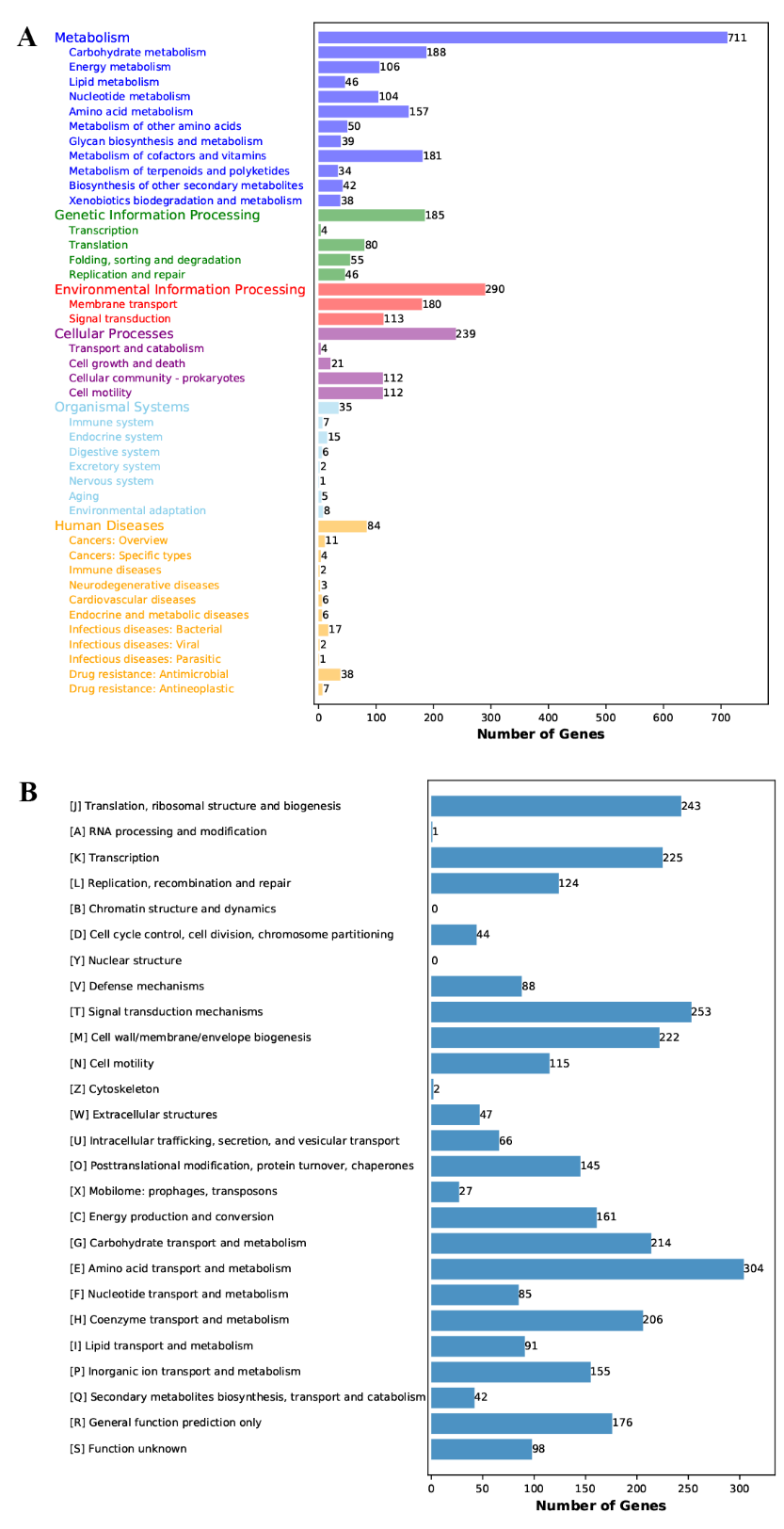


**Supplementary Figure 4**. Phylogenetic tree built from genome blast distance phylogeny (GBDP) using the type strain genome server for pairwise comparison among ZJS20^T^ and its closely related type strains. The numbers above branches are GBDP pseudo-bootstrap support values >60% from 100 replications, with average branch support of 99%.


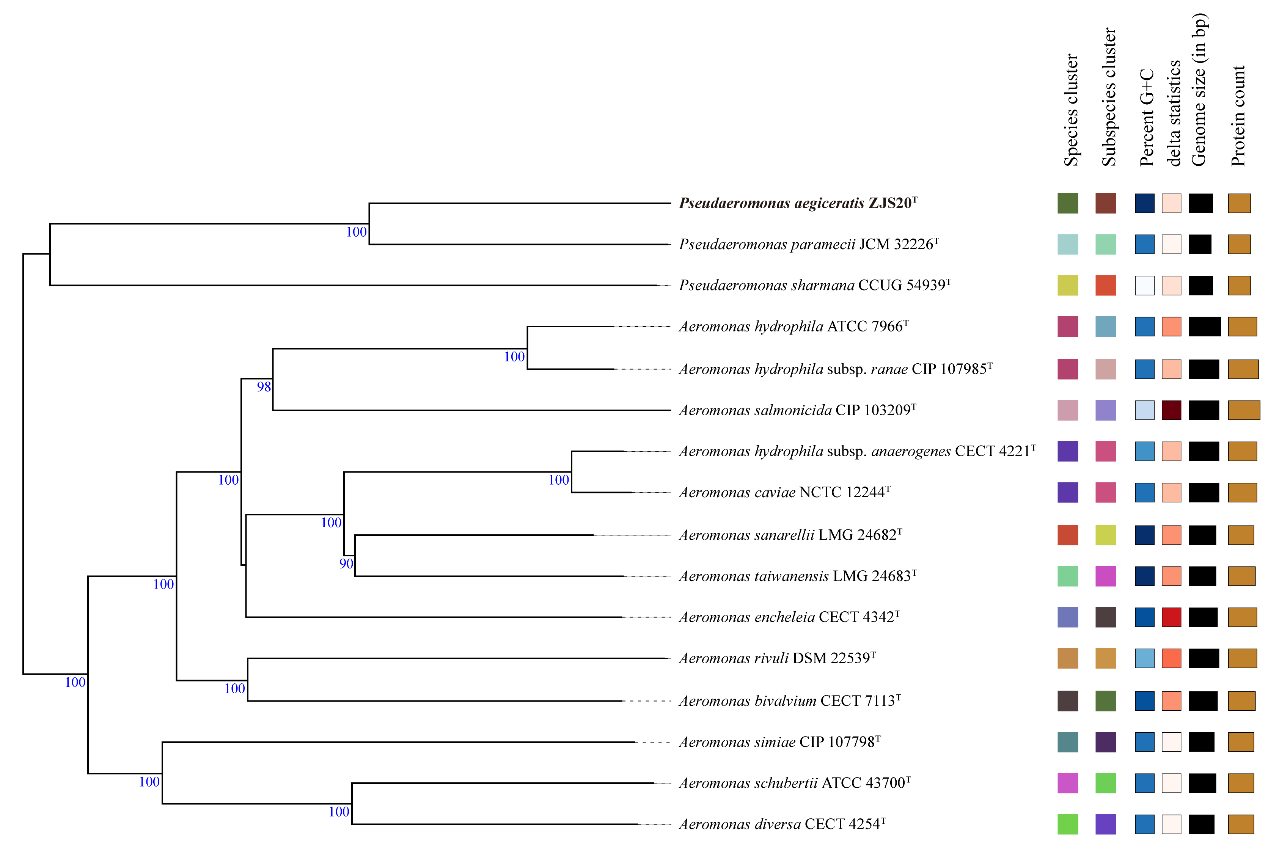

Supplement: Supplemental material — Supplemental tables and figures. [file spectrum.00041-25-s0001.docx]
